# Perioperative oxygen fraction – effect on surgical site infection and pulmonary complications after abdominal surgery: a randomized clinical trial. Rationale and design of the PROXI-Trial

**DOI:** 10.1186/1745-6215-9-58

**Published:** 2008-10-22

**Authors:** Christian S Meyhoff, Jørn Wetterslev, Lars N Jorgensen, Steen W Henneberg, Inger Simonsen, Therese Pulawska, Line R Walker, Nina Skovgaard, Kim Heltø, Peter Gocht-Jensen, Palle S Carlsson, Henrik Rask, Sharaf Karim, Charlotte G Carlsen, Frank S Jensen, Lars S Rasmussen, the PROXI Trial Group

**Affiliations:** 1 Department of Anaesthesia, Centre of Head and Orthopaedics, Copenhagen University Hospital, Rigshospitalet, Copenhagen, Denmark; 2 Copenhagen Trial Unit, Center for Clinical Intervention Research, Rigshospitalet, Copenhagen University Hospital, Copenhagen, Denmark; 3 Department of Surgery, Bispebjerg Hospital, University of Copenhagen, Copenhagen, Denmark; 4 Department of Anaesthesia, the Juliane Marie Centre, Copenhagen University Hospital, Rigshospitalet, Copenhagen, Denmark; 5 Department of Surgery, Vejle Hospital, Vejle, Denmark; 6 Department of Surgery, Copenhagen University Hospital, Herlev, Denmark; 7 Department of Surgery, Copenhagen University Hospital, Amager, Copenhagen, Denmark; 8 Department of Anaesthesia, Nykobing Falster Hospital, Nykobing Falster, Denmark; 9 Department of Surgery, Slagelse Hospital, Slagelse, Denmark; 10 Department of Anaesthesiology, Aarhus University Hospital, Aarhus, Denmark; 11 Department of Anaesthesiology, Odense Universitetshospital, Svendborg, Denmark; 12 Department of Surgery, Naestved Hospital, Naestved, Denmark; 13 Department of Surgery, Viborg Hospital, Viborg, Denmark; 14 Department of Anaesthesia, Copenhagen University Hospital, Gentofte, Denmark

## Abstract

**Background:**

A high perioperative inspiratory oxygen fraction may reduce the risk of surgical site infections, as bacterial eradication by neutrophils depends on wound oxygen tension. Two trials have shown that a high perioperative inspiratory oxygen fraction (FiO_2 _= 0.80) significantly reduced risk of surgical site infections after elective colorectal surgery, but a third trial was stopped early because the frequency of surgical site infections was more than doubled in the group receiving FiO_2 _= 0.80. It has not been settled if a high inspiratory oxygen fraction increases the risk of pulmonary complications, such as atelectasis, pneumonia and respiratory failure. The aim of our trial is to assess the potential benefits and harms of a high perioperative oxygen fraction in patients undergoing abdominal surgery.

**Methods and design:**

The PROXI-Trial is a randomized, patient- and assessor blinded trial of perioperative supplemental oxygen in 1400 patients undergoing acute or elective laparotomy in 14 Danish hospitals. Patients are randomized to receive either 80% oxygen (FiO_2 _= 0.80) or 30% oxygen (FiO_2 _= 0.30) during surgery and for the first 2 postoperative hours. The primary outcome is surgical site infection within 14 days. The secondary outcomes are: atelectasis, pneumonia, respiratory failure, re-operation, mortality, duration of postoperative hospitalization, and admission to intensive care unit. The sample size allows detection of a 33% relative risk reduction in the primary outcome with 80% power.

**Discussion:**

This trial assesses benefits and harms of a high inspiratory oxygen fraction, and the trial may be generalizable to a general surgical population undergoing laparotomy.

**Trial registration:**

ClinicalTrials.gov identifier: NCT00364741.

## Background

Surgical site infection is a common and serious complication after abdominal surgery [1]. It is essential to optimize perioperative conditions because the first hours following bacterial contamination are critical for establishing the wound infection [2]. Wound oxygen tension is often low at the end of surgery and this is unfortunate, because bacterial eradication depends on this factor *via *oxidative killing by neutrophils [3-6]. The incidence of surgical site infections may therefore be reduced by increasing the perioperative arterial oxygen tension through increased inspiratory oxygen fraction.

Before we initiated our multicenter trial "PeRioperative OXygen Fraction – effect on surgical site Infection and pulmonary complications after abdominal surgery" (PROXI), we undertook a meta-analysis on trials comparing the effect of perioperative inhaled oxygen fraction of 0.80 with 0.30 on the frequency of surgical site infection. This was performed using the trial sequential analysis method [7-9] and in accordance with international recommendations [10,11].

When searching MEDLINE, Cochrane Central Register of Controlled Trials, and EMBASE (search terms, see Appendix) four clinical trials including 1003 patients were found [12-15].

**Figure 1:**
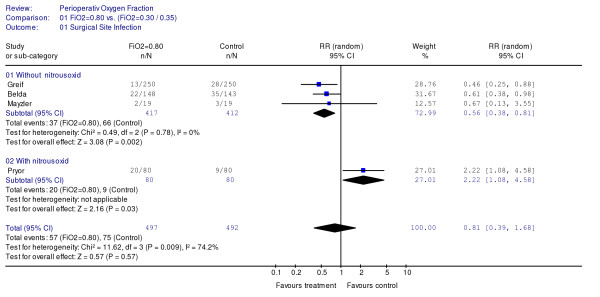
Meta-analysis comparing perioperative inspiratory oxygen fractions of 0.80 and 0.30/0.35 on surgical site infection.

In a random-effects model, the overall pooled effect of an inspiratory oxygen fraction of 0.80 was a reduction of the occurrence of surgical site infections. The relative risk reduction was 19% [95% CI: -68% to 61%], P = 0.57, but a large heterogeneity, I^2 ^= 74%, was also found (Fig. 1). This may primarily be explained by the findings in one trial [15], in which the high oxygen fraction was associated with an increase in the frequency of surgical site infection of 122% [95% CI: 8% to 458%]. In that trial, however, mixtures of oxygen and nitrous oxide were given, the surgical site infections were assessed retrospectively by chart review, and the allocation was not fully concealed.

When inspired in a high concentration, oxygen may result in pulmonary complications, but although 1003 patients have participated in the previous trials [12-15], this important question has been studied only in 30 patients [16]. This subgroup of patients from the first trial of supplemental oxygen [13] underwent pulmonary function test together with chest radiographs and computed tomography (CT) scans on the day after surgery. A high oxygen fraction was not found to be associated with significant changes in any test, but CT-determined atelectasis tended to be more common in patients receiving 80% oxygen (94% vs. 64%, P = 0.12). Preoxygenation with 100% oxygen for 5 minutes has also been associated with increased risk of atelectasis formation when compared to 60% [17]. A high oxygen fraction has also been related to harms such as an increased risk of airway inflammation [18], poor regulation of blood glucose [19], changes in the cardiac index [20], and to benefits such as improved healing of colorectal anastomosis [21] and reduced frequency of postoperative nausea and vomiting [22,23].

We designed the PROXI-Trial to assess the potential benefits and harms of a high perioperative oxygen fraction in patients undergoing laparotomy, the primary end point being surgical site infection.

## Methods and design

### Trial design

The PROXI-Trial is an ongoing, randomized, parallel group, multicenter, patient- and assessor blinded trial, launched on October 9, 2006. The trial is investigator initiated and controlled. The primary aim is to assess the effect of a high perioperative oxygen fraction on the frequency of surgical site infection in patients undergoing laparotomy. The secondary outcome measures are: atelectasis, pneumonia, respiratory failure, re-operation, mortality, duration of postoperative hospitalization, and admission to intensive care unit. Fourteen anaesthesia and surgical centres as well as one trial unit in Denmark participate in the trial.

### Inclusion criteria

Patients, aged 18 years or older, scheduled for acute or elective laparotomy are eligible for inclusion. When the laparotomy is indicated for a gynaecological disease, only patients with suspected malignancy (defined as risk of ovarian malignancy index >200 [24] or a specimen showing atypical or neoplastic cells) are included.

### Exclusion criteria

The exclusion criteria are: Surgery performed under general anaesthesia within 30 days, chemotherapy for malignancy within 3 months, inability to give informed consent, and preoperative arterial haemoglobin oxygen saturation below 90% assessed by pulse oximetry without supplemental oxygen.

### Randomization

The patients are randomized 1:1 by a central interactive voice-response system at the Copenhagen Trial Unit to ensure adequate allocation concealment. A computer generated randomization sequence with variable block size is used with the following stratification variables: Centre, diabetes mellitus, acute or elective surgery, and body mass index (<30 or ≥30 kg/m^2^).

### Intervention

After induction of anaesthesia and tracheal intubation, patients randomized into the supplemental oxygen group are given an inspiratory oxygen fraction (FiO_2_) of 0.80 until end of surgery. Patients are administered a FiO_2 _of 0.80 during the first two hours following extubation by means of a non-rebreathing face mask with a reservoir (High Concentration Oxygen Mask, Intersurgical Ltd, Wokingham, UK) with a flow of 14 litres of oxygen and 2 litres of air per minute. This mixture of oxygen and air contains a slightly higher oxygen fraction (0.901), because even the manufacturer's test resulted in only 85% delivered oxygen in a situation with masks fully sealed to a flat surface and 15 litres of oxygen per minute (Intersurgical Test Report, April 2008). With an estimated flow of ambient air into the mask of approximately 3.0 litres per minute, we estimate that the chosen mask and mixture delivers a FiO_2 _close to 0.80. This was confirmed in a test before the trial was initiated.

The patients randomized into the control group are given a FiO_2 _of 0.30 after tracheal intubation and until extubation, after which they receive a flow of 2 litres of oxygen and 14 litres of air per minute through a non-rebreathing facemask with reservoir (High Concentration Oxygen Mask, Intersurgical Ltd, Wokingham, UK).

In both groups, it is allowed to increase FiO_2 _if hypoxia is detected or suspected in order to keep the arterial oxygen saturation above 94% and the arterial oxygen tension above 9 kPa. Positive end expiratory pressure (PEEP) is used at a level chosen by the attending anaesthetist. At the end of the intervention period, oxygen is administrated only at the physician's discretion and according to usual clinical practice.

### Blinding

Cardboard shields are placed on the side of the anaesthesia machines to keep the surgical team blinded to group allocation. In the post anaesthesia care unit, opaque bags cover the flow meters. Information about perioperative FiO_2_, arterial oxygen partial pressure (PaO_2_) as well as flow of oxygen and air is collected on a separate paper form, placed in a sealed opaque envelope when patients are discharged from the post anaesthesia care unit. Any urgency requiring opening of the envelope will be reported. The patients are not informed of their group allocation during the trial or follow-up.

Patients are asked after follow-up which group they believe they were allocated to in order to evaluate patient blinding and the possible related bias in the reporting of adverse events. If patients answer supplemental oxygen or control group, they are asked to indicate why.

The Steering Committee is also blinded and has no access to patient allocation during the trial. An independent statistician will analyze the PROXI data under code (treatment A and B) and prepare a blinded version of the results. All sections of the manuscript, including the discussion and conclusion, will be written in two versions; one assuming treatment A is supplemental oxygen and treatment B is control treatment, and another manuscript based on the reverse assumption [25]. All authors must approve both versions before demasking the allocation groups.

### Standard treatment

After preoxygenation, anaesthesia is induced with propofol or thiopental supplemented with remifentanil, fentanyl, sufentanil, or alfentanil and maintained with propofol, sevoflurane, or desflurane. The use of nitrous oxide is not allowed. Tracheal intubation is facilitated with succinylcholine or an intermediate acting non-depolarizing neuromuscular blocking agent. Both groups are given a FiO_2 _of 1.0 until tracheal intubation and again immediately before extubation. The patients are ventilated to assure normocapnia (defined as an arterial carbon dioxide tension of 4.5 to 6.0 kPa if arterial blood sampling is carried out, otherwise capnography is used to adjust ventilation).

Several important elements of the perioperative care [26-30] are stressed in the trial protocol (Table 1). The protocol recommends cefuroxime 1.5 g and metronidazole 1.0 g given intravenously as standard antibiotic choice, but we define appropriate antibiotic therapy according to Table 2[31] because of the high number of surgical procedures. Antibiotic therapy must be given within 60 minutes of skin incision, and we consider 'timely administration' to be fulfilled if the first and second antibiotic is given before skin incision.

**Table 1:** Trial protocol for perioperative care of patients undergoing laparotomy.

Protocol element	Description
Preoperative:	
Bowel preparation	No routine oral preparation used for colonic resection.*
Fasting guideline	Allowed to drink clear fluids 2 hours before anaesthesia.*
Perioperative:	
Epidural analgesia	Placed at thoracic level corresponding to the incision in elective procedures and used intraoperatively.*
Fluid therapy	A preoperative deficit in acute surgery corrected preoperatively, but no routine fluid preload used. Fluid given only to replace measured or calculated deficits (no third space loss) aiming at a body weight increase less than 1 kg. Peroperative blood loss replaced 1:1 with colloids, not exceeding 500 mL more than estimated blood loss. Blood transfusion initiated if blood loss exceeds 20 mL/kg, considering the patient's haematocrit. Vasopressors or reduction of epidural infusion if hypotension.^¤^
Temperature control	Warmed fluids if large infusions and upper body air-warming device used. Core temperature measured continuously, aiming at 36 to 37°C.*
Glucose control	Aim: Blood concentration between 5 and 11 mmol/L.
Surgical technique	Shortest possible abdominal incision. No intraabdominal drain, no nasogastric tube unless essential for intraoperative gastric decompression, postoperative ileus prophylaxis or postoperative nutrition.*
Neuromuscular function	Monitored with a nerve stimulator; patients are not extubated before train-of-four ratio is above 0.90.^#^
Postoperative:	
Pain relief	Epidural analgesia continued for 2 days postoperatively. Paracetamol 4 g daily and a non-steroidal anti-inflammatory drug before discontinuing the epidural analgesia. An opioid is given intravenously if pain score at rest is above 3 on visual analogue scale (0–10).*
Fluid therapy	Oral intake as early as possible, blood loss replaced 1:1 with colloids or blood transfusion according to normal clinical practice. Other deficits replaced with crystalloids in order to keep urine output above 1 mL/kg/hr.^¤^

* Fearon et al. [26]; ^# ^Berg et al. [27]; ^¤ ^Arkilic et al., Kabon et al., Brandstrup et al. [28-30].

**Table 2:** Adequate perioperative intravenous antibiotic prophylaxes.

Type of surgery	Adequate perioperative antibiotic prophylaxis
Elective colorectal surgery	B or C
Elective gynaecological surgery	
Clean procedures	A
Clean-contaminated, contaminated or dirty infected procedures	B or C
Elective removal of gall bladder	None
Acute appendectomy, no perforation	None
Acute appendectomy, with perforation	B or C
Other acute laparotomy,	
Clean procedures	None
Clean-contaminated, contaminated or dirty infected procedures	B or C

A = cefuroxime 1.5 g; B = cefuroxime 1.5 g and metronidazole 1.0 g; C = ampicillin 2 g or benzylpenicillin 2 million IU in combination with gentamicin 0.240 g and metronidazole 1.0 g.

Adequate antibiotic prophylaxes divided by surgical procedure are based on the advisory statement from the National Surgical Infection Prevention Project [31].

### Baseline data

After inclusion we record demographic characteristics and data on significant comorbidity with emphasis on the following factors: Current smoking, ethanol consumption above 48 g daily, diabetes mellitus, concurrent infection, or immunosuppressive disease, chronic obstructive pulmonary disease (COPD) and other pulmonary diseases. The risk of infection is evaluated with the National Nosocomial Infections Surveillance System (NNISS) and the Study on the Efficacy of Nosocomial Infection Control (SENIC) scores [32,33].

### Perioperative data collection

Preoperative haemoglobin and peroperative change in blood glucose are measured. We record duration of anaesthesia, duration of surgery, placement of epidural catheter, type of anaesthesia, body core temperature at the end of surgery, and use of antibiotics, vasopressors, and dexamethasone. We record peroperative blood loss and the administered volume of crystalloids, colloids, and blood. Pre- and postoperative body weight is also measured, if possible.

### Follow-up

All patients must be seen daily in the postoperative period by a surgical investigator blinded to the allocated intervention. A follow-up visit is scheduled between the 13^th ^and the 30^th ^postoperative day as appropriate. The primary and secondary outcome measures are evaluated at each visit and additional information about wound characteristics in the postoperative period is collected to calculate the ASEPSIS score (Additional treatment, Serous exudate, Erythema, Purulent exudate, Separation of deep tissues, Isolation of bacteria and duration of inpatient Stay) [34]. This score (range 0–70) combines wound appearance the first 5 postoperative days with additional surgical treatment and a score higher than 20 indicates wound infection [34].

Patients presenting with symptoms of pulmonary complications are examined according to routine clinical practice by the attending physician, including chest radiographs or CT, when relevant. All chest radiographs and CT's are evaluated for infiltrate and atelectasis by the attending radiologist, who is unaware of the administered intervention.

### Outcome measures

The primary outcome is surgical site infection within 14 days, defined according to the criteria by Center of Disease Control and prevention (CDC) [35]. This definition includes superficial, deep, and organ/space infections and surgical site infection is considered present if any of these infections are diagnosed during follow-up. If a patient has a combination of superficial, deep, and organ/space infections, we report the deepest infection, except from organ/space infections that drain through the incision, which according to CDC is a deep surgical site infection [35].

The secondary outcomes are defined as follows (intervals defined as time after surgery):

• Pneumonia within 14 days, defined according to the criteria by CDC [36]. We will report the frequencies within this category of: Nosocomial pneumonia, ventilator-associated pneumonia, pneumonia due to gross aspiration, and pneumonia in immunocompromised patients [36].

• Atelectasis within 14 days is defined to be present if described in the radiologist's evaluation of chest radiograph or CT.

• Respiratory failure within 14 days, defined as need for controlled ventilation or arterial oxygen saturation below 90% despite supplemental oxygen.

• Mortality within 30 days.

• Duration of postoperative hospitalization, including readmission periods, if occurring within 30 days.

• Admission to the intensive care unit within 14 days, if not part of the postoperative care.

• Abdominal re-operation due to any reason within 14 days.

In case of uncertain outcome measures, two blinded assessors, and a third assessor in case of further disagreement, review the patient's hospital record.

### Adverse events

All recorded adverse events will be reported according to the CONSORT Statement [37]. We do not list any adverse events specifically related to supplemental oxygen in the protocol or consent form. The following adverse events are considered so frequent after surgery that they are not recorded: Pain or hypotension within the first 3 postoperative days and abnormal laboratory values that do not require medical treatment. All other adverse events are collected prospectively in the patient's case report form and specifically addressed at the follow-up visit. The reported adverse events will be categorized before the trial's allocation groups are demasked.

An adverse event is considered serious if it is fatal, life threatening, causing permanent disability or requiring prolonged hospitalization. Adverse events and serious adverse events will be reported for all randomized patients separately as frequencies for each arm. It will be reported if any adverse event results in increase or decrease of the allocated FiO_2_.

### Missing data

If patients do not meet for the follow-up visit, we contact: Hospital outpatient clinics, emergency departments, and the patient's family physician. Wound evaluation carried out in accordance with the CDC-criteria is considered adequate. In the remaining cases, the patients are interviewed by telephone, and the information obtained is used in the intention-to-treat analysis.

Missing data from daily evaluation of wound characteristics for the ASEPSIS score will be replaced by scores obtained by linear regression of score by day using the scores from the adjacent days. Missing data in patients discharged before 5^th ^postoperative day will be replaced by scores obtained by linear regression of score by day between the adjacent in-hospital score and the score at the follow-up visit [38].

Patients meeting the inclusion criteria without being randomized are prospectively recorded. Completeness of these data is established through the Danish Anaesthesia Database  and the Danish National Patient Registry [39] by searching the relevant Health Service Classification System (SKS)-codes for laparotomy procedures .

### Major protocol violations

Patients with the following major protocol deviations will not be included in the *per protocol *analysis: Not meeting the inclusion criteria, fulfilling an exclusion criterion, FiO_2 _above 0.60 for more than 1 hour in the control group, FiO_2 _below 0.60 for more than 1 hour in supplemental oxygen group, failure to use the oxygen mask more than 1 hour, no in-hospital evaluation of the outcomes for 4 consecutive days or more, no follow-up visit between 13^th ^and 30^th ^postoperative day, and unblinded outcome assessment. We considered the limit of FiO_2 _= 0.60 to represent the lowest oxygen fraction where atelectasis could not be attributed to the oxygen concentration [17].

### Trial conduct and monitoring

Data are collected on printed case report forms, on which a unique barcode number is printed in order to eliminate possibilities of duplication of the case report forms. Case report forms are scanned to the database using the Verity Teleform^® ^system (Verity, Sunnyvale, California, USA), which may have an even higher accuracy than manual transfer of data to an electronic database by double data entry.

### Statistics

All data will be analyzed according to a predefined plan. Only the primary and secondary outcomes and serious adverse events will be compared statistically. Outcome measures will be analyzed for all randomized patients in the intention-to-treat analysis, which will be the primary results of the trial. According to the International Conference on Harmonization Good Clinical Practice (ICH-GCP) guidelines for analyses of randomized clinical trials of medicinal products No 9 [40] univariate analyses will be carried out for all outcome measures. In multivariate analyses, the intervention effects will be adjusted by the following covariates being the stratification variables: centre, diabetes mellitus, acute or elective surgery, and body mass index (<30 or ≥30 kg/m^2^) as well as the following: COPD, daily smoking, surgical incision extending above the umbilical transversal, duration of surgery, and age (<40 or ≥40 years). All intervention effect estimates will be given with 95% confidence limits and a two-tailed P-value < 0.05 considered significant.

### Sample size

We estimated the frequency of surgical site infection to be 16% in the control group. This was based on the previously reported frequencies [12-15] and the inclusion of acute laparotomies in our trial. A fixed effects meta-analysis  model showed a relative risk reduction of 25% if all 4 trials are included  and 45% if the Pryor trial [15] were excluded. We thus expected a relative risk reduction of 33%. We calculated that a total sample size of 1400 patients would allow us to detect or reject a difference in surgical site infection between 16% and 10.7%, with 5% type 1 error risk, 80% power, and 10% dropout.

### Trial sequential analysis of cumulative meta-analysis

**Figure 2:**
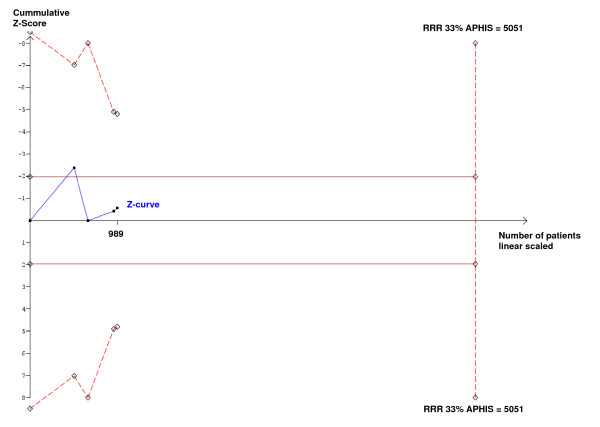
**Trial sequential analysis with a required information size of 5051**. A priori heterogeneity adjusted information size (APHIS) based on an a priori relative risk reduction (RRR) of 33% with a type I error risk of 5% and a power of 80%. The cumulative z-curve constructed for a random effects model as heterogeneity is 74% crosses the traditional boundary (P = 0.05) once and return to non-significant values. The cumulative z-curve never crosses the trial sequential monitoring boundary. Despite 989 patients randomized we may still need more than 4000 randomized participants to close the information gap considering repeated analyses of accumulating data.

**Figure 3:**
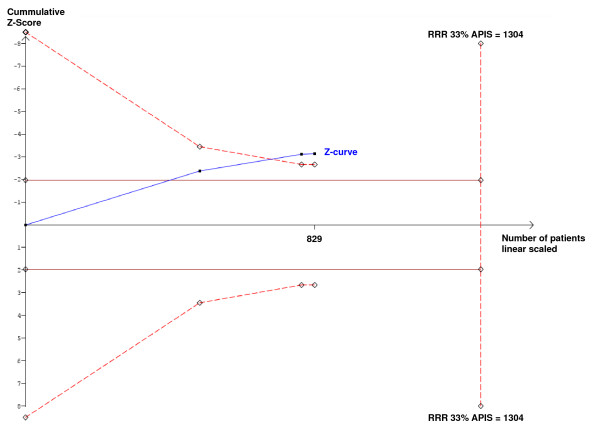
**Trial sequential analysis excluding the trial of Pryor**. Meta-analysis of the trials by Greif [13], Belda [12] and Mayzler [14], excluding the trial of Pryor [15] with a required information size of 1304 (APIS, a priori information size) based on an a priori relative risk reduction (RRR) of 33% and a type I error risk of 5% and a power of 80%. The cumulative z-curve constructed for a fixed-effect model as heterogeneity is 0% crosses both the traditional boundary (P = 0.05) after the first trial and the trial sequential monitoring boundary during the second trial. So there may be evidence for an effect of at least 33% RRR in a cumulative meta-analysis of trials investigating a high oxygen fraction when the Pryor trial is excluded when adjusting for repeated analyses of accumulating data.

In a single trial, interim analyses increase the risk of type I error. To avoid an increase of overall type I error, monitoring boundaries can be applied to decide whether a single randomized trial could be terminated early because of the P-value being sufficiently small. Because no reason exists why the standards for a meta-analysis should be less rigorous than those for a single trial, analogous trial sequential monitoring boundaries can be applied to meta-analysis as trial sequential analysis [7-9]. The underlying assumption for this analysis is that significance testing is performed each time a new trial is published. Trial sequential analysis depends on the quantification of the required information size. Cumulative meta-analysis of trials are at risk of producing random errors, because repetitive testing of accumulating data runs the risk of random errors and the information size requirement, analogous to the sample size of a single optimally powered clinical trial, is not met. Information size calculations were based on an assumption of a plausible relative risk reduction with an a priori relative risk reduction of 33% surgical site infections. The trial sequential analysis [7] adjusting for repeated testing on accumulating data shows that we still lack sufficient information dependent of the Pryor trial [15]. If all trials were included, neither the trial sequential monitoring boundary nor the traditional boundary (P < 0.05) were crossed (Fig. 2), and the required heterogeneity adjusted information size is 5051 to reliably detect or reject a relative risk reduction of 33% with a type I error risk of 5% and a type II error risk of 20%. If the Pryor trial [15] is excluded, the cumulative meta-analysis may be conclusive adjusted for repeated significance testing in cumulative meta-analysis, as the trial sequential monitoring boundary is crossed during the second trial (Fig. 3). As this post hoc exclusion of one of the trials testing FiO_2 _= 0.80 vs. FiO_2 _= 0.35 may be biased, we therefore concluded, considering the result of the meta-analysis of all the trials, that there may still be an information gap of more than a thousand randomized patients. So we calculated that 1400 patients must be randomized and assessed to reliably confirm a detection or rejection of a 33% relative risk reduction of surgical site infections after abdominal surgery with FiO_2 _= 0.80 vs. FiO_2 _= 0.30.

### Data Monitoring Committee

An independent Data Monitoring Committee (DMC) was established to evaluate safety and efficacy at one scheduled interim analysis. This took place when the first of the following three events occurred: Follow-up of the first 700 patients, ninety patients diagnosed with surgical site infection or 100 diagnosed with pneumonia. Primary and secondary outcome measures, occurrence of any serious adverse event and occurrence of any non-serious adverse event were presented to the DMC under blinded codes for the 2 arms of the trial. The DMC could advise the steering committee to stop the trial if the interim analysis demonstrated:

• Conclusive evidence of a decreased frequency of the primary outcome measure (benefit) with a high oxygen fraction, with a P-value < 0.001 against the control group.

• Conclusive evidence for increased frequency of the primary outcome measure (harm) with a high oxygen fraction, with a P-value < 0.01 against the control group.

• Number and nature of serious adverse events outweighed by any potential benefits.

The DMC recommended continuing the trial after the interim analysis held on January 24, 2008, with 563 patients analyzed as more than ninety patients had a surgical site infection at that time.

### Ethical considerations

The PROXI-Trial is conducted in compliance with the Helsinki Declaration and approved by the Research Ethics Committee of Copenhagen and Frederiksberg (protocol No H-KF-306766), the Danish Medicines Agency (protocol No 2612-3165), and the Danish Data Protection Agency (protocol No 2006-41-6738). The trial is registered at  (NCT00364741). All patients sign written informed consent before arrival to the operating room. The trial is conducted and monitored according to the ICH-GCP guidelines [41]. Case report forms are checked for validity and internal consistency through centre visits where source data are inspected.

### Trial status

In the beginning of September 2008, a total of 1350 patients are enrolled at the 14 participating centres: Rigshospitalet (n = 273), Bispebjerg Hospital (n = 152), Vejle Hospital (n = 138), Herlev Hospital (n = 128), Amager Hospital (n = 113), Nykobing Falster Hospital (n = 106), Slagelse Hospital (n = 99), Aarhus Hospital (n = 90), Svendborg Hospital (n = 79), Naestved Hospital (n = 62), Viborg Hospital (n = 57), Gentofte Hospital (n = 39), Holbaek Hospital (n = 8), Kolding Hospital (n = 6).

## Discussion

The benefits of a high perioperative oxygen fraction on surgical site infections may be substantial, but a considerable gap of information exists before this is firmly established. Furthermore, potential harms from a high oxygen fraction, primarily pulmonary complications, have not been adequately assessed.

**Figure 4:**
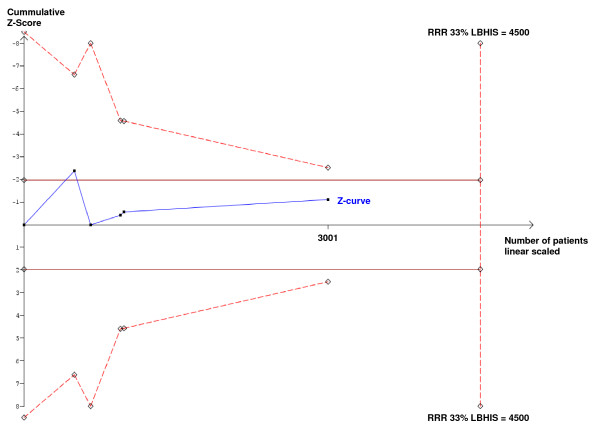
**Trial sequential analysis of all trials irrespective of adjuvant inhaled gases**. The effect of 80% oxygen vs. 30% oxygen on surgical site infections calculated in cumulative meta-analysis of all trials irrespective adjuvant inhaled gases (the trials by Greif [13], Pryor [15], Belda [12], Mayzler [14] and Myles [42]). The low-bias heterogeneity adjusted information size (LBHIS) is 4500 based on a relative risk reduction (RRR) suggested by the low-bias trials of 33% and a meta-analytic estimate of the frequency of surgical site infection in the control group (30% oxygen) on 14% with a type I error risk of 5% and a power of 80%. No crossing of the trial sequential monitoring boundary at any time despite P < 0.05 after the first trial [13]. The gap of information to reject an intervention effect of 33% relative risk reduction is approximately 1500 patients.

Some additional trials suggest that a high oxygen fraction in the perioperative period is beneficial. Firstly, patients undergoing nitrous oxide-free anaesthesia with 80% oxygen had fewer wound infections than patients receiving nitrous oxide-based (70% N_2_O, 30% oxygen) anaesthesia [42]. The higher oxygen concentration could have contributed significantly to this difference, because nitrous oxide may not be a risk factor for wound infections [43]. Secondly, another large trial investigating treatment of hypoxia via continuous positive airway pressure in the postoperative period also demonstrated a reduction in wound infections [44]. However, even if the nitrous-oxide trial [42] is incorporated in the trial sequential analysis, there is still a gap of information of approximately 1500 patients to reject an intervention effect of 33% relative risk reduction (Fig. 4).

### Strengths

Our trial is the first trial to report potential benefits as well as harms in all patients receiving supplemental oxygen. This is strengthened as a consequence of the mandatory monitoring according to the ICH-GCP standards [41], including adverse events.

The low-bias design [45] and large sample size allows us to reliably detect even smaller intervention effects than the four previous trials [12-15]. We furthermore report surgical site infections according to the CDC-criteria [35], which also consider the most severe surgical site infection; the organ/space infection.

In addition, the PROXI-Trial is the first investigation of supplemental oxygen including acute patients. Apart from higher rates of peroperative contamination, these patients may have more cardiovascular and pulmonary comorbidity than elective patients and accordingly a higher risk for low local tissue oxygen partial tension, which could increase the benefit of a high oxygen fraction. On the other hand, these patients are also more prone to postoperative pulmonary complications and a potential harm of a high oxygen fraction cannot be excluded either.

We choose to include gynaecological cancer surgery, because this account for a large and increasing proportion of laparotomies, as increasing numbers of colorectal procedures are now performed laparoscopically. We believe this may strengthen the external validity of the trial and the generalizability of the trial results. Patients with benign gynaecological conditions are not considered for inclusion. This was decided because we primarily sought to include patients with a high risk of surgical site infections, thus avoiding low power to detect or reject an intervention effect. The frequency of surgical site infection is estimated to be only 2% in patients with American Society of Anesthesiologists physical status score I-II undergoing clean or clean-contaminated abdominal hysterectomy with a duration of surgery less than 2 hours [1] and 4% after abdominal hysterectomy for benign conditions [46].

The stratified randomization is used to avoid skewed allocation of patients with important prognostic factors for surgical site infection and allows us to adjust intervention effect estimates for the stratification variables (diabetes mellitus, obesity and acute surgery) with the highest power. The lack of such stratification was a major limitation in the trial by Pryor et al. [15]. With stratification for center, we furthermore match the different distribution of surgical procedures in the participating hospitals.

### Limitations

Some important limitations must be noted. Firstly, some patients in the control group may need more than the allocated 30% oxygen in order to keep arterial oxygen saturation above 94%. However, this practice is in accordance with clinical practice and we believe such pragmatic nature of the intervention is important. Our *per protocol *analysis will assess if close adherence to the protocol is associated with better outcome.

Secondly, we are not able to apply all elements of the standard treatment to all patients. Timely administration of antibiotics and epidural analgesia influences postoperative outcome, but this is not possible to achieve in all patients. The antibiotic regimen is recorded to assess whether it is adequate for the given type of surgery and the most common pathogenic bacterial flora. Protocol deviations may result in a higher frequency of surgical site infection, but that may reflect clinical practice.

Thirdly, it is possible that the mixture of different surgical procedures may be associated with the risk of overlooking a beneficial effect related to specific surgical procedures, such as colorectal resections, but the type of surgery is not always known at the time of deciding the inspiratory oxygen fraction and we are aiming at elucidating the effect of a high oxygen fraction in connection with open gastroenterological procedures in general, including emergency surgery.

## Conclusion

We believe our pragmatic trial design increases the external validity, because the protocol is in accordance with clinical practice. We anticipate that the results of this trial may be generalizable to a general surgical population undergoing laparotomy.

## List of abbreviations

APHIS: a priori heterogeneity adjusted information size; APIS: a priori information size; ASEPSIS: additional treatment, serous exudate, erythema, purulent exudate, separation of deep tissues, isolation of bacteria and duration of inpatient stay; CDC: Center of Disease Control and prevention; CONSORT: consolidated standards of reporting trials; COPD: chronic obstructive pulmonary disease; CT: computed tomography; DMC: data monitoring committee; FiO_2_: inspiratory oxygen fraction; ICH-GCP: International Conference on Harmonization Good Clinical Practice; LBHIS: low-bias heterogeneity adjusted information size; NNISS: National Nosocomial Infections Surveillance System; PaO_2_: arterial oxygen partial pressure; PEEP: positive end expiratory pressure; PROXI: Perioperative Oxygen Fraction – Effect on Surgical Site Infection and Pulmonary Complications after Abdominal Surgery: a Randomized Clinical Trial; RRR: relative risk reduction; SENIC: Study on the Efficacy of Nosocomial Infection Control; SKS: health service classification system codes.

## Competing interests

The authors declare that they have no competing interests.

## Authors' contributions

Each author has made substantial contributions to the conception and design and has been involved in the critical revision of the manuscript for important intellectual content. Specifically, CSM is the principal coordinating investigator and have drafted the manuscript together with JW. LNJ, SWH, IS, TP, LRW, NS, KH, PGJ, PSC, HR, SK, CGC, and FSJ are principal site investigators and have coordinated the enrolment or follow-up visits at their centre. LSR is the sponsor. Additionally, CSM, JW, LNJ, and LSR are members of the steering committee. All authors read and approved the final manuscript.

## Appendix

 A non-language restricted search string for PUB MED search of randomized trials for the effect of perioperative supplemental oxygen for surgical site infection.

"Surgical" [Text Word] AND "infection" [Text Word] AND "oxygen" [Text Word] AND "Randomized Controlled Trial" [ptyp] AND "adult" [MeSH Terms] AND "hominidae" [MeSH Terms].

Initial search February 2006, last update September 2008.
